# OA29 Paediatric Osteomyelitis Following Bacillus Calmette-Guérin Vaccination: A Case Report

**DOI:** 10.1093/rap/rkae117.029

**Published:** 2024-11-05

**Authors:** Marwa Mohareb, Kapil Garg, Nagui Gendi

**Affiliations:** Mid and South Essex Hospitals, Basildon, United Kingdom; Mid and South Essex Hospitals, Basildon, United Kingdom; Mid and South Essex Hospitals, Basildon, United Kingdom

## Abstract

**Introduction:**

Mycobacterium tuberculosis (TB) is a major cause of morbidity and mortality. Prior to the COVID-19 pandemic, TB infection was the leading cause of death globally.

Children under 5 are particularly susceptible, as indicated by 2022 UK notifications. The Bacillus Calmette-Guérin (BCG) vaccine, a live attenuated immunization, is not part of the routine NHS vaccination schedule but is recommended for those at higher risk, such as children with a parent or grandparent from high TB-risk countries. BCG-related systemic complications are rare. The most common complication is BCG lymphadenopathy, followed by osteomyelitis that may develop 4-24 months post vaccination.

**Case description:**

We are reporting a case of BCG osteomyelitis in a paediatric patient without evidence of systemic manifestations.

In April 2018, a 2-year-old British boy whose mother is of Indian descent, presented to the orthopaedic department with non-traumatic persistent left wrist swelling noticed by his parents for three weeks. Examination revealed swelling and limited extension and flexion at 10 degrees. No other joint affection.

There was no history of psoriasis, constitutional manifestations, or eye problems, and there was no travel history. Additionally, he was up to date with his immunizations.

X-rays and ultrasound were inconclusive for bony or joint abnormalities but showed marked tenosynovitis of the extensor tendons, suggestive of reactive synovitis/arthritis, subsequently he was referred to the rheumatology department.

Rheumatological examination confirmed synovitis of the left wrist. Blood tests revealed normal inflammatory markers and negative rheumatoid factor, anti-CCP, and ANA. Intraarticular injection under local anaesthetic cream provided temporary relief but symptoms recurred, the patient had been diagnosed with pauciarticular juvenile arthritis.

**Discussion:**

Repeat ultrasound showed tenosynovitis of the 1st and 2nd dorsal compartments. Lucency in the metaphysis with soft tissue swelling, loss of cortical definition is demonstrated to the distal radial epiphysis was seen on the X ray.

An urgent MRI showed ring enhanced lesion in left distal radial metaphysis with extension across the physis to epiphysis. There was significant tenosynovitis and focal collection dorsally.

As there was no evidence of systemic disease and rest of joints were normal, starting in metaphysis with extension across the physis to epiphysis, osteomyelitis was the most likely diagnosis.

The distal radius epiphysis was drilled and irrigated. A bony window was created in the metaphysis, revealing a significant amount of pus, which was promptly debrided. The area was then filled with Cerament G and the bone window was closed. Finally, the wrist was immobilized in a backslab for 2 weeks. The patient received IV ceftriaxone and oral fusidic acid. Histopathological analysis confirmed mycobacterium TB on PCR (mycobacterium bovis vaccine strain). Further history revealed that the child received BCG vaccines at 5 months old.

Anti-tubercular therapy was started including isoniazid (switched to moxifloxacin due to transaminitis), ethambutol, rifampicin, pyridazole and clarithromycin for six months. Six weeks post-surgery, the patient showed favourable progress with no evidence of collection or inflammation around the left distal radius, no constitutional manifestation or lymphadenopathy.

Two months later, an MRI showed significant inflammation reduction but persistent abnormality in the bone marrow of the distal radius, mostly reparative processes. After six months of treatment, X-rays showed satisfactory progress and MRI revealed normal appearance without evidence of collection. The child was followed up until the age of 5 without recurrence.

Normal wrist function was regained, with no tenderness or swelling, leading to discharge from the rheumatology clinic. Extensive investigation ruled out immunodeficiency and genetic causes.

**Key learning points:**

• Diagnosing BCG osteomyelitis is challenging due to its nonspecific symptoms and the delay between vaccination and symptom onset. While there are no specific guidelines for treatment, previous literature suggests that antituberculosis regimens, particularly isoniazid and rifampicin, are effective. As BCG osteomyelitis is rare, particularly in immunocompetent children, diagnosing it can be time-consuming. Therefore, we should be aware of this potential complication to facilitate diagnosis and prevent confusion with pyogenic osteomyelitis.

• This case highlights the significance of taking complete vaccination history and ethnicity when evaluating persistent non-traumatic joint swellings, even without typical constitutional symptoms or elevated inflammatory markers. Early detection is crucial to prevent bone or joint damage

